# Recombinant protein expression in *Acanthamoeba castellanii*

**DOI:** 10.3389/fbioe.2025.1524405

**Published:** 2025-03-20

**Authors:** Pooja Salunke, Kiran Kondabagil, Yogesh A. Karpe

**Affiliations:** ^1^ Agharkar Research Institute, Nanobioscience Group, Pune, India; ^2^ Savitribai Phule Pune University, Pune, India; ^3^ Department of Biosciences and Bioengineering, Indian Institute of Technology Bombay, Mumbai, India

**Keywords:** recombinant expression, protozoa, glycoprotein, post transcription/translation modifications, protein expression and purification

## Abstract

The ongoing quest to improve protein production efficiency, quality, and versatility fuels the exploration of novel expression systems. In this research, we explored the potential of the axenically culturable Acanthamoeba as an alternative for producing recombinant eukaryotic proteins. We constructed plasmid vectors utilizing the TBP promoter to facilitate recombinant protein expression within this protozoan system. Our primary objectives were to develop an efficient transfection method and assess the capacity of *Acanthamoeba castellanii* for glycoprotein expression. Our initial efforts yielded successful expression of the firefly luciferase reporter gene, allowing us to optimize the transfection protocol. Subsequently, we compared the expression of the Chikungunya virus E2 protein across three systems: *E. coli*, Acanthamoeba, and mammalian cells. Interestingly, the E2 protein expressed in Acanthamoeba exhibited a molecular weight higher than bacterial cells but lower than mammalian cells, suggesting the possibility of glycosylation occurring in the protozoan system. These findings collectively suggest that protozoa, like *A. castellanii*, represent a promising avenue for developing low-cost and efficient eukaryotic expression systems.

## 1 Introduction

Protein expression systems are required to produce large quantities of specific proteins for research, biotechnological, and medical applications. These systems often involve the use of organisms like bacteria, yeast, insect cells, and mammalian cells. The choice of system depends on the protein’s complexity and desired modifications. Bacterial systems are relatively fast and inexpensive but may not perform the complex folding and post-translational modifications needed for some proteins (Greven and Brett, 2022; Pouresmaeil and Azizi-Dargahlou, 2023). Post-translational modifications are pivotal in regulating protein function and biological processes (Ramazi and Zahiri, 2021). They modulate the activity of a wide array of eukaryotic proteins (Ramazi and Zahiri, 2021). The complexity of proteins, especially those requiring multiple post-translational modifications, poses challenges in their expression (Shabalina et al., 2013). Eukaryotic systems, like yeast or mammalian cells, can handle these complexities but are significantly costlier for large-scale protein production (Kesidis et al., 2020; Samuel Guemra, 2014). The limitations in existing expression systems have led to the development of alternative systems capable of modifying post-translational modifications in recombinant proteins (Thoring et al., 2019). The constant search for better expression systems focuses on balancing efficiency, cost, and the ability to produce functional proteins with appropriate post-translational modifications. Therefore, this study aims to explore protozoa as a potential protein expression system.

Protozoa are unicellular eukaryotes, and Acanthamoeba is a genus of microscopic, single-celled protozoa belonging to the amoeba group. They are found commonly in diverse habitats like soil, freshwater, and marine environments (Dinda et al., 2024; Nagyová et al., 2010). Acanthamoeba can undergo encystation, which allows them to survive in harsh conditions (Wang et al., 2023). While mostly non-pathogenic in environmental settings, certain Acanthamoeba species can cause opportunistic infections in immunocompromised individuals. One such disease is *Acanthamoeba* keratitis, a severe eye infection caused by this organism (Anaísa et al., 2022). *Acanthamoeba castellanii* serves as a model organism for studying cell-cycle regulation, cytoskeleton movement, and phagocytosis (Siddiqui and Khan, 2012; Wang et al., 2023. *Acanthamoeba castellani* is also a popular host for isolating giant viruses, serving as a bridge in understanding virus-host interactions (Chatterjee et al., 2016; Chatterjee et al., 2019; Robert et al., 2004). Moreover, Acanthamoeba is recognized for its role as a host for various microorganisms, including viruses, bacteria, protozoa, and yeast (Siddiqui and Khan, 2012). Acanthamoeba species are essential for assessing cellular virulence in pathogenic bacteria, aiding in understanding environmental bacterial infections (Leoni Swart et al., 2018).

Protozoa, such as *A. castellanii*, have a sophisticated protein expression system that can carry out post-translational modifications, such as glycosylation and the formation of disulfide bonds (Garate et al., 2004). These modifications, especially N-linked glycosylation, play a significant role in protein folding and stability, influencing the overall conformation and function of proteins (Garate et al., 2004). Non-pathogenic Acanthamoeba is a promising host for recombinant protein expression due to its safety, cost-effectiveness, and ease of cultivation (Garate et al., 2004).

Research on the recombinant protein expression in Acanthamoeba has highlighted several promoters, including TBP, GAPDH, and TPBF, for successful gene expression (Bateman, 2010). In a study by Bateman (2010), stable transfection was achieved in Acanthamoeba castellanii using TBP and CSP21 gene promoters, enabling the production of a TBP-EGFP fusion protein along with a neomycin resistance gene (Bateman, 2010). This work provides insights for enhancing transfection methods, potentially enabling RNAi gene knockdown and stable gene integration in the Acanthamoeba genome (Peng et al., 2005).

Transfection methods in Acanthamoeba such as electroporation (Hu and Henney, 1997) and chemical reagents like Viafect™ (Promega) (Rolland et al., 2020) and SuperFect^®^ (Qiagen) have traditionally shown low efficiency (Moon et al., 2009; Peng et al., 2005). A recent study has demonstrated that polyethylenimines (PEI), which are cationic polymers, a more affordable and accessible option, can enhance transfection efficiency (Anaísa et al., 2022). These advancements enable transient and stable transfections, allowing researchers to examine protein function and cellular localization, enriching studies on Acanthamoeba biology and potential pathogenicity (Kong and Pollard, 2002).

While previous studies explored reporter genes and protein expression in Acanthamoeba, none have investigated glycoprotein expression within this system. Our study specifically explores the potential of *A. castellanii*, a non-pathogenic and axenically culturable protozoan, as a eukaryotic platform for glycoprotein expression. Acanthamoeba’s complete eukaryotic machinery and the recent advancements in transfection techniques make it a compelling candidate for a novel protein expression system, particularly for complex glycoproteins.

## 2 Material and methods

### 2.1 *Acanthamoeba castellanii* culture and mammalian cell culture

*Acanthamoeba castellanii* (ATCC strain; 30010), a non-pathogenic organism, was used as the host for this study. Cells were cultivated and routinely grown axenically in PYG medium (20 g Protease Peptone, 1 g yeast extract, 0.5 mM CaCl_2_, 4 mM MgSO_4_, 2.5 mM KH_2_PO_4_, 2.5 mM Na_2_HPO_4_, 2 M Glucose/1,000 mL) with pH 6.5 containing Antibiotic Antimycotic (ABAM) solution (HiMedia) at 25°C. Cell counts were done with a hemocytometer.

Mammalian cell line Huh7 S10-3 (Subclone of a human liver cell line) was obtained from Dr. Suzanne U. Emerson, NIH, Bethesda, MD. Cells were maintained in Dulbecco’s modified Eagle’s medium (DMEM) with GlutaMAX (Invitrogen) supplemented with 10% fetal bovine serum (FBS) (Invitrogen) and Antibiotic Antimycotic (ABAM) solution (HiMedia). They were maintained at 37°C in a humidified incubator with 5% CO_2_.

### 2.2 Development of protozoan expression vectors

#### 2.2.1 Development of expression vector with TBP promoter from *A. castellani* and firefly luciferase reporter gene

**FIGURE 1:**
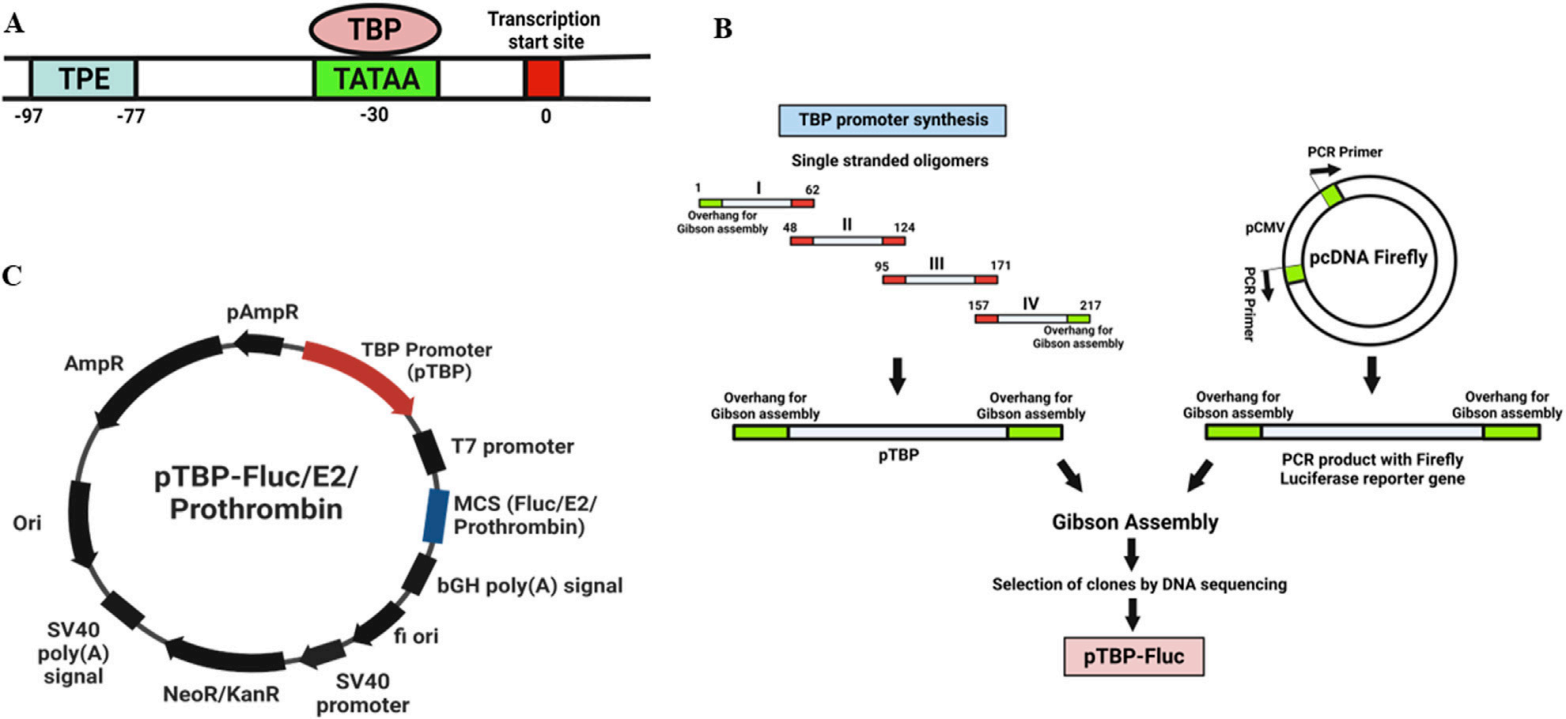
**(A)** Schematic representation of a core TBP promoter region. The transcription start site (TSS) is marked at position 0 (red box). The TATA box (light green) is located approximately 30 base pairs upstream, where the TATA-binding protein (TBP) binds to initiate transcription. The transcriptional proximal element (TPE) is situated between −97 and −77 base pairs (blue box). **(B)** Cloning strategy used for the development of Acanthamoeba Expression vector. The TBP promoter (177 bp) (Figure 1A) was synthesized by sequential annealing of four linear oligonucleotides (Supplementary Table S1: Primers 1–4). pcDNA firefly, a mammalian expression vector containing a CMV promoter and firefly luciferase reporter gene (previously cloned in our lab), served as the vector backbone. The CMV promoter was removed from pcDNA firefly vector by PCR (Supplementary Table S1: Primers 5–6), and replaced with the TBP promoter (Supplementary Table S1: Primers 7–8) using Gibson Assembly (NEB). Plasmids were isolated using the alkaline lysis method. Clones were verified using restriction digestion, TBP PCR, and DNA sequencing. This construct was designated as a pTBP-Fluc vector. **(C)** Maps of Expression vectors used in this study. Plasmid contains TBP promoter with Firefly luciferase reporter gene/Chikungunya virus E2 gene/prothrombin gene in multiple cloning site (MCS).

The TBP promoter (177 bp) (Figure 1A) was synthesized by sequential annealing of four linear oligonucleotides (Supplementary Table S1: Primers 1–4). pcDNA firefly, a mammalian expression vector containing the CMV promoter and firefly luciferase reporter gene (previously cloned in our lab), served as the vector backbone. The CMV promoter was removed from pcDNA firefly vector by PCR (Supplementary Table S1: Primers 5–6), and replaced with the TBP promoter (Supplementary Table S1: Primers 7–8) using the Gibson Assembly (NEB). The cloning strategy is schematically represented in Figure 1B. Plasmids were isolated using the alkaline lysis method. Clones were verified using restriction digestion, TBP PCR (Supplementary Table S1: Primers 13–14), and DNA sequencing. This construct was designated as a pTBP-Fluc vector (Figure 1C).

#### 2.2.2 pTBP-E2 vector construction for Chikungunya virus (CHIKV) E2 protein expression

An expression vector was developed to express an N-terminal Myc-tagged Chikungunya virus E2 protein in Acanthamoeba cells. Chikungunya virus (NCBI Genbank accession number EF027134.1) RNA was isolated using a viral RNA/DNA extraction kit (Qiagen) and reverse transcribed into cDNA using the abm OneScript reverse transcriptase cDNA synthesis kit (Applied Biological Materials Inc.). The E2 gene was then amplified from the cDNA with overhangs complementary to the vector for Gibson Assembly (Supplementary Table S1: Primers 9–10). The pTBP-Fluc vector served as the backbone (Supplementary Table S1: Primers 11–12). The Firefly luciferase reporter gene under the TBP promoter was replaced with the Chikungunya virus E2 gene using Gibson Assembly (NEB). This construct was designated as a pTBP-E2 vector (Figure 1C).

#### 2.2.3 pTBP-prothrombin vector construction for prothrombin protein expression

A similar strategy was used to develop an expression vector with another glycoprotein and TBP promoter. To investigate the expression of a high molecular weight glycoprotein (M.W. 72 kDa) in Acanthamoeba cells, the prothrombin gene was cloned under the TBP promoter. Prothrombin, also known as factor II, is a crucial protein in blood clotting (hemostasis). This construct was designated as pTBP-prothrombin (Figure 1C).

### 2.3 *A. castellanii* transfection by SuperFect reagent

In this study, Transfection conditions were optimized using the SuperFect reagent to enhance the expression of the pTBP-Fluc plasmid, which drives firefly luciferase expression in *A. castellanii* cells. Logarithmic phase trophozoites were used for transfection, and key parameters such as transfection medium, DNA concentration, and cell density were systematically optimized to maximize transfection efficiency.

For pTBP-Fluc vector transfection, Acanthamoeba cells (1 × 10^6^ trophozoites) were harvested by centrifugation at 3000 RPM for 5 min at RT. The cells were resuspended in 24 mL PYG medium and seeded into two 12-well plates (1 mL/well) for duplicate experimental sets. Plates were incubated overnight at 25°C. The next day, transfection was carried out when cells reached 70%–80% confluency. Two micrograms of supercoiled pTBP-Fluc plasmid DNA (3–6 μL volume) were diluted in either 75 μL encystment medium (Set 1) or 75 μL PYG medium (Set 2). Fifteen microliters of SuperFect reagent (Qiagen) were added to each dilution, mixed gently by pipetting and incubated for 15 min at RT.

The transfection setup included controls: nuclease-free water (no plasmid), a pcDNA firefly vector with the mammalian CMV promoter and firefly luciferase reporter gene, a PGL4 vector without a promoter, and a pUbgx vector with a ubiquitin promoter and EGFP reporter gene. An additional control received only encystment or PYG medium without reagents.

The old media were removed from all wells. Following the 15-minute incubation, this DNA-SuperFect mixture along with 600 μL of encystment medium (Set 1) or PYG medium (Set 2) was added to each well to reach a final volume of 700 μL. Transfected cells were incubated overnight at 25°C. The next day, the transfection media were replaced with 1 mL of fresh PYG medium. Cells from wells 1-6 of were harvested 24 h post-transfection, while cells from wells 7–12 were harvested 48 h post-transfection. Then washed with PBS and resuspended in 150 μL of 1x passive lysis buffer (Promega) for luciferase assay.

#### 2.3.1 Luciferase activity measurement

The total protein concentration in each sample was measured by a Bicinchoninic Acid (BCA) assay. Ten micrograms of protein from each sample were used for the luciferase assay. Firefly luciferase activity was then assessed using the Luciferase Assay System (E1500; Promega) with 50 μL LARII substrate on a luminoskan system (Thermo Scientific).

### 2.4 Transfection of pTBP-E2 plasmid in *A. castellanii* cells

Since protozoa can potentially produce recombinant glycoproteins, we decided to test the expression of recombinant Chikungunya virus (CHIKV) E2 glycoprotein in this system. The choice of CHIKV-E2 protein was based on its well-established glycosylation profile and known characteristics. The CHIKV-E2 protein has two N-glycosylation sites located at positions 263 and 345 (Acharya et al., 2015). *A. castellanii* cells were transfected with pTBP-E2 plasmid using SuperFect reagent (Qiagen) as described previously. Notably, only an encystment medium was used as the transfection medium in this experiment. Cells were harvested 48 h post-transfection. Then washed with PBS and resuspended in 150 μL of 1x passive lysis buffer (Promega) for luciferase assay.

### 2.5 Western blot analysis of Chikungunya virus E2 (CHIKV-E2) protein

Cell lysates from pTBP-E2 transfected Acanthamoeba cells containing 20 μg of protein, were then mixed with 2X Laemmli dye (SDS-PAGE reducing buffer) (10 mL of 0.625 M Tris (pH 6.8), 2 g SDS, 10 mL Glycerol, 5 mL β-Mercaptoethanol, 0.01 g Bromophenol Blue, H_2_O added to a final volume of 50 mL), heated at 95°C for 10 min, and subsequently resolved on a 12% SDS-PAGE and then electro blotted onto a PVDF membrane. Membranes were probed with a CHIKV-E2-specific primary antibody (16A12; Native Antigen Company) at a 1:4,000 dilution. Following primary antibody incubation, the membranes were washed with PBST and probed with Anti-Mouse IgG-HRP (#31430; Invitrogen) conjugated secondary antibody (1:20,000 dilution). The membranes were developed with ECL substrate (Chemiluminescent peroxidase Substrate-3, Sigma) and analyzed on a Syngene Chemi-doc system.

### 2.6 Confirmation of CHIKV-E2 gene expression by PCR

Total RNA was extracted from pTBP-E2 transfected Acanthamoeba cells using the Trizol reagent, according to the manufacturer’s protocol (ThermoFisher Scientific). CHIKV cDNA was synthesized according to previously described procedure and utilized as a positive control.

To detect and confirm CHIKV-E2 gene expression, PCR was performed using E2 gene-specific primers (Supplementary Table S1: Primers15-16) to amplify a 400-base pair (bp) segment of the cDNA. The primer sequences and PCR conditions used for the E2 gene amplification are given in Supplementary.

Control PCR was conducted using RNA as the template instead of cDNA to ensure the absence of plasmid carryover in the PCR reactions. PCR products were analyzed using agarose gel electrophoresis using a 1% agarose gel. The gels were visualized to confirm the expected 400 bp amplification product of the E2 gene.

### 2.7 Transfection of *Acanthamoeba castellanii* cells with human prothrombin gene containing plasmid

Further, we aimed to investigate the expression of a glycoprotein with a higher molecular weight in this system. We chose the prothrombin protein (72 kDa) as our target due to its well-known glycoprotein nature and thoroughly characterized N-glycosylation pattern.

The plasmid pTBP-prothrombin encoding prothrombin protein was transfected into Acanthamoeba cells using the SuperFect reagent as described previously. Immunoblotting was carried out using a 1:5,000 dilution of anti-thrombin polyclonal antibody (# PA5-99213; Invitrogen).

### 2.8 Transfection of pVax-E2 and pcDNA-Prothrombin plasmid in Huh7 cells

Huh7 cells were used for the transfection of pVax-E2 and pcDNA-Prothrombin plasmids due to their hepatocyte origin, which supports liver-specific protein expression and post-translational modifications. Additionally, Huh7 cells are permissive for CHIKV replication, making them ideal for studying both CHIKV E2 protein expression and prothrombin. We have extensively used this cell line for the expression of these two proteins in the context of host-virus interactions in our lab. Huh7 (1 × 10^6^) cells were harvested and centrifuged at 3000 RPM for 5 min at 4 °C, then washed with ice-cold phosphate-buffered saline and resuspended in 0.4 mL of ice-cold Opti-MEM™ medium (Invitrogen). Eight micrograms of plasmid DNA were added to the cells and incubated on ice for 15 min. Electroporation was performed using a Gene Pulser Xcell system (Bio-Rad) with an exponential decay pulse at 200 V and 975 μF in a 4 mm electroporation cuvette. Immediately post-electroporation, cells were transferred into a Dulbecco’s Modified Eagle Medium (DMEM) (HiMedia) medium with 20% fetal bovine serum (Invitrogen). The culture medium of transfected cells was replaced with DMEM with 10% fetal bovine serum and ABAM solution after 24 h. Cells were maintained at 37 °C with 5% CO_2_ and then harvested 48 h post-transfection.

### 2.9 Recombinant production of CHIKV-E2 protein in *E. coli*

The pET-28b vector (69865–3; Addgene) containing the CHIKV-E2 gene was used to transform the competent *E. coli* BL21 (Rosetta) variant by heat shock. The bacteria were cultured in Luria–Bertani (LB) broth containing 50 μg/mL kanamycin until the optical density reached 0.4. Protein expression was then induced by adding 1.0 mM IPTG and incubating for 4 h at 37°C. Following induction, bacterial cell pellets were collected and analyzed for protein expression using 12% SDS-PAGE, followed by immunoblotting using Anti CHIKV-E2 antibody (NAC).

## 3 Results

### 3.1 Expression of firefly luciferase reporter gene in *Acanthamoeba castellanii*

**FIGURE 2:**
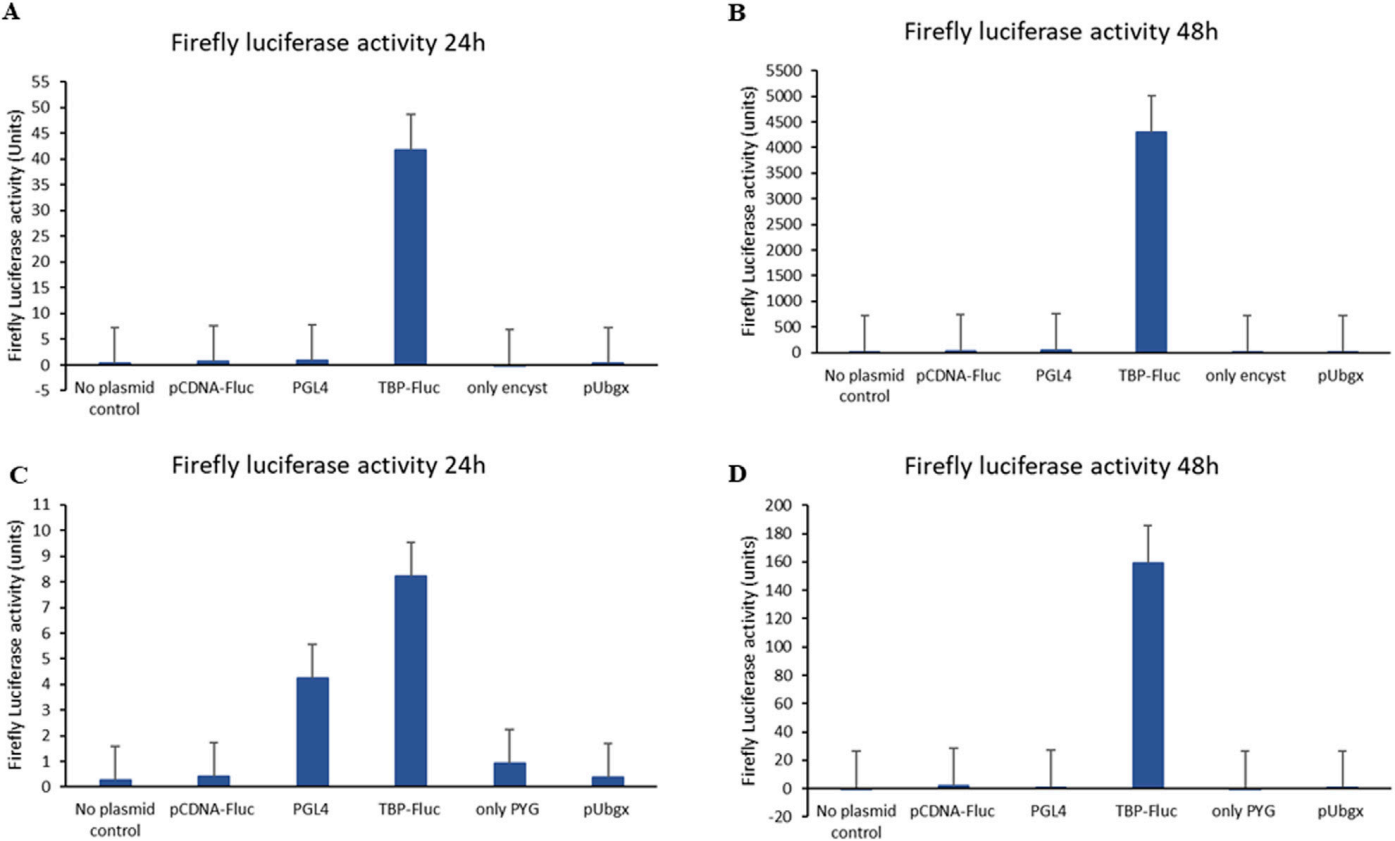
Firefly luciferase activity of Acanthamoeba cells after transfection with pTBP-Fluc plasmid and other control plasmids. Acanthamoeba trophozoites (1 × 10^6^ cells) were transfected with the pTBP-Fluc plasmid using SuperFect reagent in either encystment medium (Set 1) or PYG medium (Set 2). Cells were harvested 24 and 48 h post-transfection, followed by firefly luciferase activity measurement. The graph shows relative firefly luciferase activity (normalized to protein concentration) for cells transfected in encystment medium versus PYG medium, at both 24 and 48-hour time points. Data represent the mean ± standard deviation of triplicate experiments. **(A)** Graph showing firefly luciferase activity of cells after 24 h of transfection when encystment medium was used as transfection medium. **(B)** Graph showing firefly luciferase activity of cells after 48 h of transfection when encystment medium was used as transfection medium. **(C)** Graph showing firefly luciferase activity of cells after 24 h of transfection plasmid when PYG medium was used as transfection medium. **(D)** Graph showing firefly luciferase activity of cells after 48 h of transfection plasmid when PYG medium used as transfection medium.

Successful transfection was achieved using the encystment medium and PYG medium as transfection media. We observed no significant difference in recovery rates of cells post transfection in both media types. Only morphological changes were observed in cells which were grown in the encystment medium. Remarkably, utilizing an encystment medium resulted in significantly higher firefly luciferase activity in cells transfected with the pTBP-Fluc plasmid compared to cells transfected with other controls at both 24 h and 48 h post-transfection (Figures 2A,B). Similarly, employing a PYG medium as a transfection medium also led to significantly increased firefly luciferase activity in cells transfected with the pTBP-Fluc plasmid compared to controls at 24 h and 48 h post-transfection (Figures 2C,D).

Specifically, firefly luciferase activity in cells transfected with the pTBP-Fluc plasmid using an encystment medium as a transfection medium reached approximately 42 units after 24 h (Figure 2A) and approximately 4,300 units after 48 h (Figure 2B). In contrast, when PYG was used as the transfection medium, firefly luciferase activity reached about 8 units after 24 h (Figure 2C) and about 160 units after 48 h (Figure 2D).

Transfection efficiency in *A. castellanii* cells was approximately 26-fold higher in the encystment medium compared to PYG medium. Using the TBP promoter, firefly luciferase expression was successfully achieved in *A. castellanii* cells.

### 3.2 Expression of Chikungunya virus E2 glycoprotein in *Acanthamoeba castellanii*

The plasmid pTBP-E2, encoding the CHIKV-E2 protein, was transfected into Acanthamoeba cells using SuperFect reagent with encystment medium, specifically selected for its high transfection efficiency. Cell lysates were collected 48 h post-transfection. These cell lysates were resolved using 12% SDS-PAGE, followed by immunoblotting using CHIKV E2-specific primary antibody (Native Antigen Company).

**FIGURE 3:**
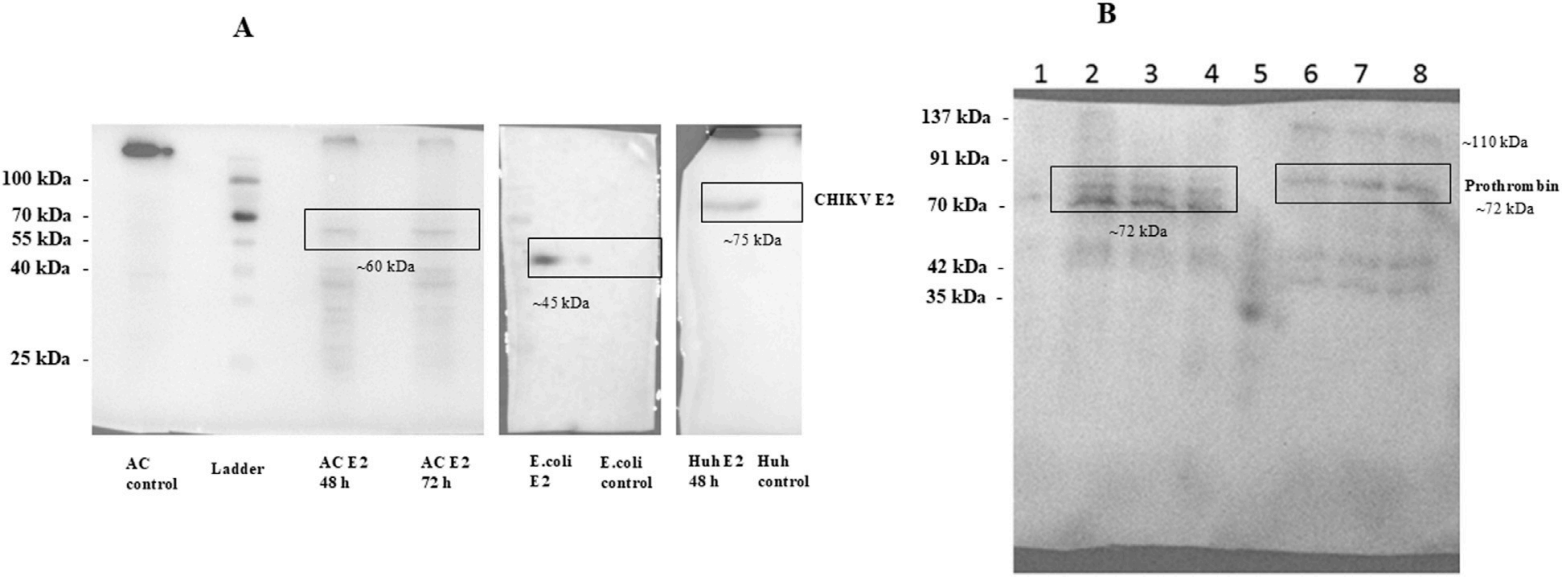
**(A)** Western blot analysis of CHIKV E2 protein expression. Expression vectors such as pTBP-E2, pET28-E2 and pVax-E2 were transfected in *Acanthomeba castellani* cells, *E.coli* cells and Huh7 cells respectively. Transfected cell lysates were collected 48 h post-transfection. These cell lysates were resolved on 12% SDS-PAGE followed by immunoblotting using CHIKV-E2-specific primary antibody (Native Antigen Company). Figure shows the expression of CHIKV E2 glycoprotein *A. castellani* cells, *E.coli* cells and Huh7 cells. **(B)** Immunoblot of prothrombin protein expressed in Acanthamoeba cells and mammalian cells. *A.castellani* cells were transfected with the plasmid pTBP-prothrombin using the SuperFect reagent, while Huh7 cells were transfected with the plasmid pcDNA-prothrombin via electroporation. Cells were harvested 48 h post-transfection, and the cell lysates were resolved on a 12% SDS-PAGE gel, followed by immunoblotting with an anti-thrombin polyclonal antibody (Invitrogen). The lanes are labeled as follows: Lane 1 represents empty plasmid transfected into Acanthamoeba cells; Lane 2 and Lane 3 show pTBP-prothrombin plasmid transfected into Acanthamoeba cells using an encystment and a PYG medium respectively, harvested 48 h post-transfection; Lane 4 shows pTBP-prothrombin plasmid transfected into Acanthamoeba cells, harvested 72 h post-transfection; Lane 5 contains the protein ladder; Lane 6 shows empty pcDNA plasmid transfected into Huh7 cells; Lane 7 shows pcDNA-prothrombin plasmid transfected into Huh7 cells, harvested 48 h post-transfection and Lane 8 represents pcDNA-prothrombin plasmid transfected into Huh7 cells, harvested 72 h post-transfection. We could detect the expression of prothrombin protein using anti-thrombin polyclonal antibody in *A.castellani* cells and Huh7 cells.

Our results revealed the expression of the CHIKV-E2 protein in *A. castellenii* cells using the specific antibody (Figure 3A). Importantly, Western blot analysis revealed a CHIKV E2-specific band with a molecular weight (∼60 kDa) slightly higher compared to the CHIKV E2 protein produced in bacterial systems (∼45 kDa) but lower than the CHIKV E2 protein expressed in mammalian cells (∼75 kDa) (Figure 3A). This observation suggests the differences in post-translational modifications between these expression systems and the possibility of partial glycosylation or other post-translational modifications such as lipidation ubiquitination, *etc.*, occurring in the protozoan system.

### 3.3 PCR amplification of CHIKV-E2 gene expressed in *Acanthamoeba castellanii*

**FIGURE 4:**
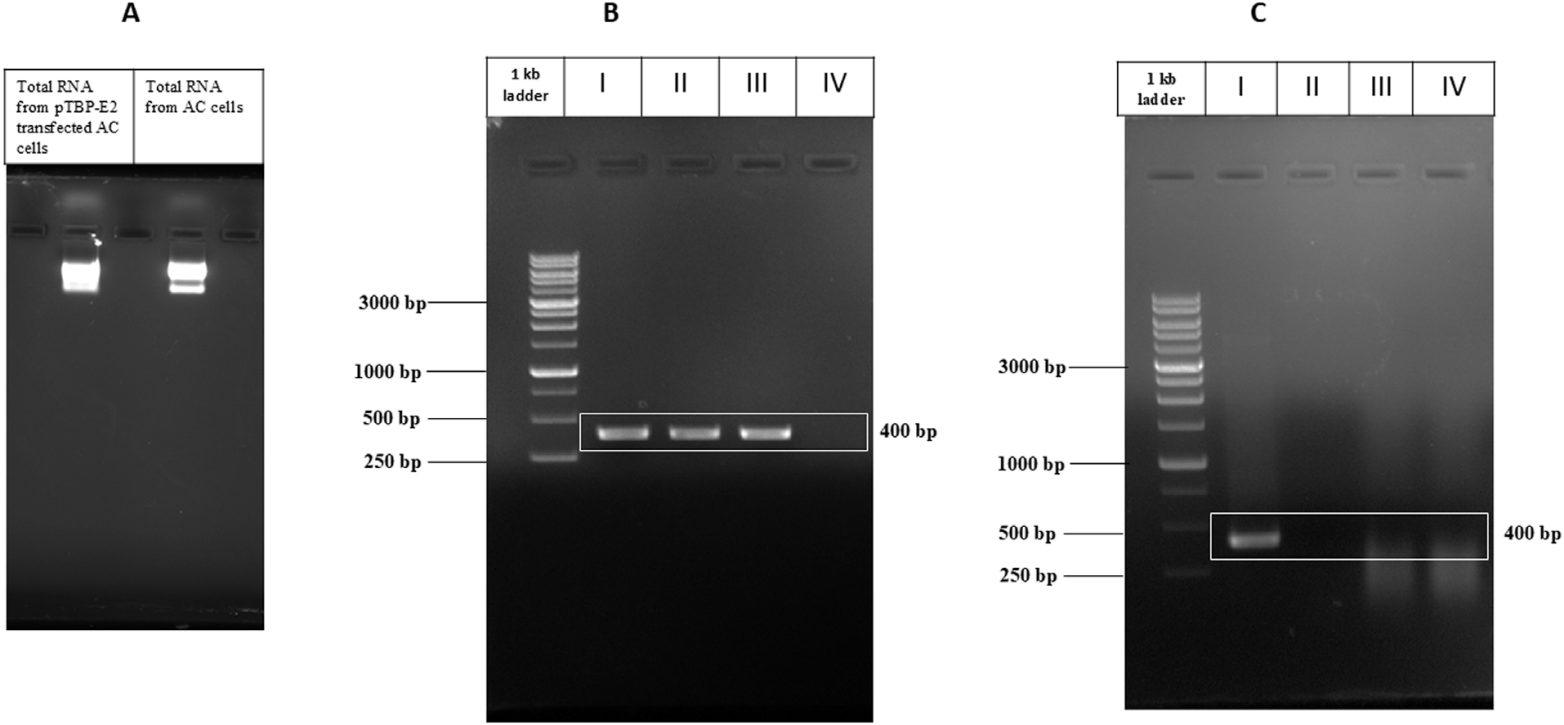
PCR Amplification of CHIKV E2 Gene. **(A)** Total RNA isolated from pTBP-E2 transfected Acanthamoeba cells and control Acanthamoeba cells. **(B)** Amplified E2 PCR products from I) pVax-E2 plasmid (PCR control), II) CHIKV cDNA, III) cDNA from Acanthamoeba cells transfected with pTBP-E2 plasmid, IV) cDNA from control Acanthamoeba cells. **(C)** Amplified E2 PCR products from I) pVax-E2 plasmid (PCR control), II) CHIKV RNA, III) RNA from Acanthamoeba cells transfected with pTBP-E2 plasmid, IV) RNA from Acanthamoeba cells.

Total RNA was successfully isolated from pTBP-E2 transfected *A. castellenii* cells using the Trizol method. The quality of the isolated RNA was evaluated through agarose gel electrophoresis indicating high-quality RNA (Figure 4A). Similarly, the CHIKV RNA extracted was confirmed to be of high quality and used as a positive control.

The mRNA reverse transcription to cDNA was successfully carried out. PCR amplification using E2 gene-specific primers yielded a single band corresponding to the expected 400 bp fragment observed in the lanes containing cDNA from pTBP-E2 transfected Acanthamoeba cells and the positive control CHIKV RNA-derived cDNA (Figure 4B). No amplification was observed in PCR with cDNA of untransfected Acanthamoeba cells. E2 amplified from the pVax-E2 clone was used as a positive PCR control. A distinct 400 bp product, confirming the expression of the E2 gene in the pTBP-E2 transfected Acanthamoeba cells. The specificity of the amplification was verified by the absence of any nonspecific bands on the agarose gel electrophoresis.

Control PCR was performed directly on RNA samples without reverse transcription to eliminate the possibility of plasmid carryover. No amplification products were observed in these control reactions (Figure 4C), confirming that the detected 400 bp PCR product was derived from cDNA and not from any contaminating plasmid DNA.

These results collectively validate the successful transfection of Acanthamoeba cells with pTBP-E2 and the subsequent expression and detection of the E2 gene through PCR amplification.

### 3.4 Expression of prothrombin in *Acanthamoeba castellanii* and Huh7 cells

Further, we aimed to investigate the expression of a glycoprotein with a higher molecular weight in this system. We chose the prothrombin protein as our target due to its well-known glycoprotein nature and thoroughly characterized N-glycosylation pattern.

The plasmid pTBP-prothrombin encoding prothrombin protein was transfected into Acanthamoeba cells using the SuperFect reagent, while pcDNA-prothrombin encoding prothrombin protein was transfected into Huh7 cells by electroporation as described previously. Huh7 cells were chosen because they are liver-derived and naturally support the expression of liver-specific proteins like prothrombin. We have previously optimized protein blots in this cell line, making it well-suited for our study. Cells were harvested 48 h post-transfection. Subsequently, cell lysates were processed for 12% SDS-PAGE, followed by immunoblotting using an anti-thrombin polyclonal antibody.

We could detect the expression of prothrombin protein using an anti-thrombin polyclonal antibody (Figure 3B). The theoretical molecular weight of prothrombin protein is 70 kDa. We observed multiple bands of the prothrombin protein, possibly reflecting its various glycosylated forms, including thrombin. Huh7 cells exhibited bands of prothrombin, including higher molecular weight forms representing fully or highly glycosylated prothrombin (∼110 kDa and ∼72 kDa), as well as bands of thrombin protein (∼35 kDa and ∼42 kDa). A similar band pattern was observed in our earlier study of Thrombin in Huh7 cells (Kanade et al., 2018). In contrast, in *A. castellanii* cells, three distinct bands of prothrombin were detected (one band at ∼72 kDa and two bands between 60-70 kDa), possibly corresponding to different partially glycosylated forms of the protein. Notably, bands corresponding to the thrombin protein were absent in Acanthamoeba cells. These results demonstrate the successful expression of the prothrombin glycoprotein in Acanthamoeba cells, underscoring the potential of this system for the production of glycoproteins.

## 4 Discussion

### 4.1 Promoters of Acanthamoeba

Promoters are essential in regulating gene expression in organisms. Protozoa exhibit remarkable diversity in their promoter design and regulatory mechanisms. In the case of *A. castellanii*, several studies have explored the use of different promoters for protein expression within this organism. These include the Acanthamoeba TBP gene promoter, TATA-binding protein promoter binding factor (TBF), glyceraldehyde phosphate dehydrogenase (GAPDH) gene promoter, Acanthamoeba cyst-specific protein 21 (CSP21) promoter, and polyubiquitin gene promoter. The TBP promoter and TPBF, studied by Wong and Bateman (1994) and Liu and Bateman (1993), respectively, regulate TBP gene expression, with TPBF acting as both an activator and repressor depending on cellular needs (Chen and Bateman, 2000; Huang and Bateman, 1995; Huang and Batemant, 1997).

The Glyceraldehyde Phosphate Dehydrogenase (GAPDH) gene promoter, is frequently used to achieve constitutive expression, including for the AcSir2 gene, demonstrating its utility for various genetic studies in *A. castellanii* (Joo et al., 2020). Furthermore, EGFP has been constitutively expressed in stably transfected *A. castellanii* by employing the promoters for the Acanthamoeba TPBF and GAPDH genes (Bateman, 2010). In contrast, the CSP21 promoter supports inducible gene expression, becoming active during encystment, making it ideal for targeting genes active in differentiation processes (Chen et al., 2004).

Another powerful promoter, the polyubiquitin gene promoter, isolated and sequenced by Hu et al., in 1997, is approximately 2.5 times more effective than the viral RSV-LTR promoter at driving gene expression in Acanthamoeba (Hu and Henney, 1997).

### 4.2 Glycosylation pattern in acanthamoeba

Glycosylation is a fundamental post-translational modification process where sugar molecules are attached to proteins, lipids, or other organic molecules. Acanthamoeba, an opportunistic human pathogen, exhibits a unique glycosylation pattern, essential in numerous biological processes, including cell adhesion, signaling, and immune evasion. Researchers characterized the N-glycome of Acanthamoeba, revealing a distinctive repertoire of glycan structures that contribute to Acanthamoeba’s biology and host-pathogen interactions. Acanthamoeba possesses all ER glycosyltransferases necessary for N-glycosylation, along with unique fucosyl- and pentosyltransferases in the Golgi (Schiller et al., 2012). The glycan composition includes oligomannosidic structures with up to four α1,2-mannose residues and 13 hexose residues, closely aligning Acanthamoeba’s N-glycan assembly with other eukaryotes like slime molds, yeast, plants, and animals, compared to other parasitic protozoans (Schiller et al., 2012). Glycosylated adhesins, such as mannose-binding and laminin-binding proteins, facilitate host attachment, key to infection processes (Lorenzo-Morales et al., 2015).

Understanding Acanthamoeba’s glycosylation patterns may facilitate the development of novel therapeutic strategies against Acanthamoeba infections (Schiller et al., 2012).

### 4.3 Other protozoan system

Protozoa such as Plasmodium, Trypanosoma, and Leishmania species exhibit complex glycosylation patterns that differ from their hosts, possessing unique monosaccharide residues, oligosaccharide linkages, and glycosylphosphatidylinositol (GPI)-anchored oligosaccharides (Guha-Niyogi et al., 2001; Willment, 2022). These patterns are essential for parasite survival and transmission, as in Leishmania, where lipophosphoglycans facilitate parasite attachment and release in the vector’s midgut, critical for transmission to humans (Sacks et al., 2000). Protozoa have the enzymatic machinery needed for protein glycosylation, (Vaccaro, 2000), which aids in host cell invasion, as seen in Cryptosporidium parvum (Paluszynski et al., 2014).

The Trypanosomatidae family, including Leishmania, has been used to express eukaryotic proteins due to their mammalian-like glycosylation (Niimi, 2012). *Leishmania tarentolae is* known for its consistent N-glycosylation pattern with biantennary complex-type oligosaccharides, making it valuable for recombinant protein production with complex post-translational modifications (Klatt et al., 2019). Due to advances in genetic manipulation and cultivation methods, the German company Jena Bioscience developed an expression system in 2002 called LEXSY (Leishmania expression system). This system efficiently produces functional, glycosylated proteins, closely mimicking human N-glycosylation, though lacking sialyltransferase (de Oliveira et al., 2019).

Additionally, Toxoplasma gondii, independently transfers both endogenous truncated and host-derived N-glycans onto its proteins, highlighting the diversity of glycosylation mechanisms among protozoa (Garénaux et al., 2008).

### 4.4 Summary of results

The results of our study indicate that *A. castellanii* holds significant potential as an alternative eukaryotic expression system for recombinant protein production, particularly for glycoproteins. Using the TBP promoter, we successfully developed vectors and optimized transfection methods to enhance gene expression in this protozoan. Optimization of transfection parameters using the SuperFect reagent, including transfection medium, DNA concentration, and cell density, led to significant improvements in transfection efficiency. Initial transfection with the firefly luciferase reporter gene demonstrated efficient expression, particularly when using an encystment medium. Our findings suggest that the encystment medium serves as a superior transfection medium for *A. castellenii* cells, resulting in a remarkable increase in the transfection efficiency of approximately 26-fold compared to the PYG medium. This success facilitated the expression of more complex proteins, such as the Chikungunya virus E2 glycoprotein and human prothrombin. The safety and efficacy of a recombinant protein can be significantly influenced by its glycosylation, which is intricately connected to its biological function. The E2 protein exhibited a higher molecular weight in Acanthamoeba compared to bacterial systems, but lower than mammalian cells. Molecular weight of E2 protein expressed in insect cells (Sf9 cells) is 55 kDa (Cho et al., 2008), which is slightly lower or comparable to the molecular weight of the E2 protein expressed in Acanthamoeba cells. This suggests the possibility of partial glycosylation or other post-translational modifications such as lipidation, ubiquitination, etc. occurring in the protozoan system. While we could see the expression of slightly higher molecular weight CHIKV E2 protein than the bacterial system, we could not obtain enough quantity of purified protein for characterizing the post-translational modification by mass spectrometry. Future efforts will be directed to improve the protein yield. Similarly, the expression of the prothrombin protein, known for its characterized N-glycosylation pattern, was confirmed in Acanthamoeba cells. These findings collectively suggest that *A. castellanii*, with its complete eukaryotic protein expression machinery, can be a cost-effective and versatile host for producing functional glycoproteins, offering an advantageous alternative to traditional systems like *E. coli* and mammalian cells. The use of protozoan promoters, characterized by both conventional and unconventional regulatory elements, further highlights the adaptability and potential of this system for diverse biotechnological applications. This study opens novel opportunities for utilizing protozoa in recombinant protein production, particularly for proteins requiring post-translational modifications such as N-type glycosylation.

## 5 Conclusion

*Acanthamoeba castellanii* emerges as a promising eukaryotic expression platform, combining the advantages of eukaryotic post-translational machinery with cost-effectiveness and ease of cultivation. This study establishes the path for further investigation and optimization of protozoan systems for biotechnological and pharmaceutical applications, potentially offering a viable alternative to existing protein expression systems.

## Data Availability

The raw data supporting the conclusions of this article will be made available by the authors, without undue reservation.

## Abbreviations

AC, Acanthamoeba castellanii; GFP, green fluorescent protein; CHIKV, Chikungunya Virus; BCA, Bicinchoninic Acid; TBP, TATA Binding Protein; GAPDH, Glyceraldehyde Phosphate Dehydrogenase; TBPF, TBP promoter-binding factor; TPE, TBP promoter element.
