# Supplementary material for: Recombinant protein expression in Acanthamoeba castellanii
Source: Front Bioeng Biotechnol. 2025 Mar 20;13:1524405. doi: 10.3389/fbioe.2025.1524405 (PMC11965365; doi:10.3389/fbioe.2025.1524405)
Supplement: Supplementary file 1 [file DataSheet1.docx]

Supplementary Data:

**Table 1: Primers used in the development of expression vectors/ PCR:**

| Sr. No. | Primer Name | Oligo sequence | Construct |
| --- | --- | --- | --- |
| 1. | TBP_1-62 | AGCCCATATATGGAGTTCCGAACGACGCCTTGCAACAAGCTGAGAAAAAACCAGGATCGGCA | **pTBP** |
| 2. | TBP_48-124 | ACAAAAAAATTGGCCCCTTTTTATACCCGGTGATGCCAACGTTTCCGTTGAAAATCCTTCCTTGCCGATCCTGGTTT | **pTBP** |
| 3. | TBP_95-171 | CCGGGTATAAAAAGGGGCCAATTTTTTTGTTGATTTGTTGCGCGAATTCTTGCTTTCGGCATCGAATTCAAGGGAGA | **pTBP** |
| 4. | TBP_157-217 | ATCTGACGGTTCACTAAACCCTTGTTGTATGTGTGAATCGACTCCTTCTCCCTTGAATTCG | **pTBP** |
| 5. | pcDna excl CMV _fwd | GAGACCCAAGCTGGCTAG | **pTBP-Fluc** |
| 6. | pcDna excl CMV _rev | AACGCGTATATCTGGCCC | **pTBP-Fluc** |
| 7. | insert_TBP_fwd | acgggccagatatacgcgttAAACGACGCCTTGCAACAAG | **pTBP-Fluc** |
| 8. | insert_TBP_rev | cgctagccagcttgggtctcCTTGTTGTATGTGTGAATCGACTC | **pTBP-Fluc** |
| 9. | E2pcDNAgibson_F | GTGCTGGATATCTGCAGAATTCACCATGGCCCCATACTTAGCTCACTGTCCCG | **pTBP-E2** |
| 10. | E2pcDNAgibson_R | CAAGCTTGGTACCGAGCTCGGATCCGCAGCATATTAGGCTAAGCAGGAAAG | **pTBP-E2** |
| 11. | pCDNA_977 FWD | GGATCCGAGCTCGGTACCAAGCTTGGG | **pTBP-E2** |
| 12. | PcDNA_959_REV | GAATTCTGCAGATATCCAGCACAGTGG | **pTBP-E2** |
| 13. | TBP−110_F | AAACGACGCCTTGCAACAAG | _ |
| 14. | TBP+68_R | CTTGTTGTATGTGTGAATCGAC | _ |
| 15. | E2_Fwd_300 | GGGATTCACTGACAGTAGGAAG | _ |
| 16. | E2_Rev_706 | CAAGTTCAGCATTACGCGGGAC | _ |

**PCR conditions used for the CHIKV-E2 gene amplification:**

The PCR conditions were optimized (Phusion enzyme) as follows: initial denaturation at 98°C for 30 seconds, followed by 30 cycles of denaturation at 98°C for 10 seconds, annealing at 63°C for 30 seconds, and extension at 72°C for 20 seconds, with a final extension at 72°C for 5 minutes.
